# Goblet cell carcinoid of the appendix: Two case reports and a review of the literature

**DOI:** 10.3892/mco.2020.2064

**Published:** 2020-06-09

**Authors:** Alejandro Livoff, Noam Asna, Enrique Gallego-Colon, Aner Zeev Daum, Tattiana Harkovsky, Moshe Schaffer

Mol Clin Oncol 11:493-497, 2019; DOI: 10.3892/mco.2019.1921

Following the publication of this paper, the authors have realized that the first and second authors on this paper (Alejandro Livoff and Noam Asna) contributed equally to this study, and therefore should have been credited as being joint first authors. Therefore, the authors' affiliations and footnotes on this paper should have appeared as follows:

ALEJANDRO LIVOFF^1*^, NOAM ASNA^2*^, ENRIQUE GALLEGO-COLON^1^, ANER ZEEV DAUM^1^, TATTIANA HARKOVSKY^2^ and MOSHE SCHAFFER^2,3^

Departments of ^1^Pathology and ^2^Oncology, Barzilai Medical Center, Ashkelon 30604; ^3^Faculty of Health Sciences, Ben Gurion University, Be'er Sheva 8410501, Israel

^*^Contributed equally

The authors all agree to this correction. The authors regret this error in the affiliations, and apologize for any inconvenience caused.

